# Extended infusion of piperacillin–tazobactam versus intermittent infusion in critically ill egyptian patients: a cost-effectiveness study

**DOI:** 10.1038/s41598-022-12861-7

**Published:** 2022-06-27

**Authors:** Christina Medhat Naiim, M. M. Elmazar, Nagwa A. Sabri, Naglaa S. Bazan

**Affiliations:** 1 Clinical Pharmacy Department, Faculty of Pharmacy, The British University in Egypt (BUE) Cairo, P.O.BOX 43, Cairo, 11837 Egypt; 2 Clinical Pharmacy Department, Faculty of Pharmacy, Ain Shams University, Cairo, Egypt; 3 Critical Care Medicine Department, Cairo University Hospitals, Cairo University, Cairo, Egypt; 4 Pharmacy Practice and Clinical Pharmacy Department, Faculty of Pharmacy, Future University in Egypt, Cairo, Egypt

**Keywords:** Diseases, Health care

## Abstract

Extended infusion of piperacillin/tazobactam over 4 h has been proposed as an alternate mode of administration to the 30-min intermittent infusion to optimize treatment effects in patients with gram-negative bacterial infections. The study aimed to evaluate the extended infusion regimen of piperacillin/tazobactam in standings of efficacy, safety, and cost to the intermittent one in the treatment of gram-negative bacterial infections. A prospective randomized comparative study was performed on 53 patients, 27 in the intermittent infusion group and 26 in the extended infusion group. The primary outcome was the mean number of days to clinical success and the percentage of patients who were clinically cured after treatment. The secondary outcomes included mortality, readmission within 30-days, and cost-effectiveness analysis based on the mean number of days to clinical success. The clinical success rate was comparable in the two groups. Days on extended infusion were significantly lower than intermittent infusion (5.7 vs 8.9 days, respectively, p = 0.0001) as well as days to clinical success (4.6 vs 8.5 days, respectively, p = 0.026). The extended infusion was superior to the intermittent infusion regarding cost-effectiveness ratio ($1835.41 and $1914.09/expected success, respectively). The more cost-effective regimen was the extended infusion. Both regimens had comparable clinical and microbiological outcomes.

## Introduction

Nowadays the most common cause of hospitalization is bacterial infections that are increasingly causing nosocomial infections as well in the critical care setting. The management of these bacterial infections is becoming more challenging due to the emergence of antibiotic resistance and the limited available treatment options^1^. Gram-negative infections (GNI) caused by bacteria such as *Entero-bacterales, Pseudomonas aeruginosa, and Acinetobacter species* have special characteristics associated with significant mortality, morbidity, and health care costs. These organisms are capable of up-regulating or attaining genes responsible for coding mechanisms that cause antibiotic drug resistance, particularly due to antibiotic selection pressure^2,3^. The emergence of resistant pathogens happens throughout the treatment of these gram-negative infections which is getting harder to be well managed. The inappropriate antimicrobial therapy evolving all the time is the reason behind the worse outcomes for patients with resistant pathogens as proved by many studies^4,5^.

Focusing to achieve improvements specifically in the treatment efficacy and patient outcomes, researchers are working on optimizing the pharmacodynamics and pharmacokinetic factors of presently available antimicrobial agents^5^. Extended or continuous modes of administration of antibiotics have been recently applied to cause the time percentage of the free drug concentrations to remain above the minimum inhibitory concentration increase (ft > MIC) and consequently, the patient outcomes may improve theoretically. In the late 1970s, when continuous infusions were first employed, this led to an increase in clinical success rates of antibiotics. However, because of several factors such as the stability, compatibility of the drugs, and limited intravenous access, they weren’t extensively applied until lately^6,7^.

Piperacillin tazobactam, a time-dependent antibiotic, is a combination of beta lactam-beta lactamase that is broadly used to treat serious gram-negative healthcare-associated infections^5,8^. As the time percent that the antibiotic levels are above the MIC has a direct relationship with the effectiveness, extended infusion of piperacillin/tazobactam is increasingly recommended these days, instead of the authorized 30-min intermittent mode of administration, to improve treatment of infections due to multi-resistant bacteria and those for which the MIC of antibiotic is high^9^.

Some studies that were closely monitoring the effect of using EI of time-dependent antibiotics, including piperacillin/tazobactam as empiric therapy for the treatment of gram-negative bacterial infections in general and especially in critically ill patients who have sepsis, found that both the conventional II and the EI dosing almost have the same rates of treatment success, mortality and even the hospital length of stay^7,10,11^. On the other hand, other studies postulated the extended or continuous infusion regimen of piperacillin/tazobactam instead of the conventional intermittent one to be used in practice due to its higher clinical success and lower mortality rates found after treatment^4,12^.

In the middle east especially in the low- and middle-income countries, no studies clinically and economically comparing the use of extended or continuous infusion strategy versus the intermittent strategy of piperacillin/tazobactam were found. We sought to determine whether an extended infusion strategy will be more efficient, safe, and cost-effective compared to the conventional strategy of intermittent regimens in Egyptian critically ill patients.

## Materials and methods

### Aim of the study

Conducting a cost-effectiveness analysis to examine the practice of using extended infusion piperacillin-tazobactam dosing strategy *vs* intermittent infusion dosing in critically ill Egyptian patients with suspected or proven bacterial infections.

### Ethical approval

The Research Ethics Committee for experimental and clinical studies at the Faculty of Pharmacy, Ain Shams University-Cairo-Egypt (REC-ASU number 198) and the council of Critical Care Medicine Department, Cairo University revised and approved this research. The principles of the Declaration of Helsinki 2013 were applied to this study^13^. Following the Basic & Clinical Pharmacology & Toxicology policy for experimental and clinical studies, this study was conducted^14^. The present study applied CONSORT guidelines and was registered at http://www.clinicaltrials.gov (NCT04895657). Date: 20/5/2021. Before participation, all guardians of eligible patients were informed about the study protocol and they provided the written informed consent.

### Study design and population

A prospective non-blinded randomized comparative study was conducted at the Critical Care Medicine Department-Cairo University Hospitals. A set of 53 patients were enrolled from the Intensive Care Unit-Cairo University Hospitals. All adults that were critically ill admitted to the Critical Care Medicine Department with suspected or proven bacterial infections on admission or during their ICU stay were evaluated for inclusion in the study. The duration of the study was 1 year and 6 months (July 2018–December 2019). Based on the local antimicrobial guideline at the Critical Care Medicine Department, piperacillin/tazobactam was considered among the first-line empirical therapy for suspected gram-negative bacteria from different sites of infection.

Patients were included if any of the following criteria were met:

- Adult patients aged between 18 and 74 years
- Patients who received piperacillin–tazobactam therapy for at least 48 h (concomitant antimicrobial therapy was allowed).
- Patients diagnosed with suspected or confirmed gram-negative bacterial infections (e.g., intra-abdominal infection, community or hospital-acquired infections of the lung, wound, skin or soft tissue, and various other infections).
- Expected ICU stay more than 24 h

Patients were excluded if any of the following criteria were met:

- Known hypersensitivity or allergy to B-lactam antibiotics.
- Pregnant or nursing patients.
- Patients documented with severe renal dysfunction (CrCl < 20 ml/min or on dialysis).
- Cancer patients.
- During the study period, if patients were admitted several times, we include the first admission only in the final analysis (others were excluded).

### Randomization

Randomization of patients into two groups in a 1:1 ratio was done using a computer-generated randomization list. To achieve allocation concealment, patients were randomized using sequentially numbered, opaque sealed envelopes (SNOSE)^15^. The randomization sequence was generated by an independent statistician. Physicians and patients were aware of the treatment allocation.

Based on the manufacturer label patients were randomly assigned to either of two groups; group 1 received piperacillin/tazobactam as an intermittent infusion (over 30 min.) every 8 h and group 2 received it as an extended infusion (over 4 h) every 8 h as well. without loading doses. Dose adjustments were made in patients with CrCl from less than 100 to 20 ml/min^16,17^.

### Data collection

Patients’ medical records and electronic files on the hospital system are used to extract from them demographic and clinical data at baseline and periodically thereafter until the day of stopping antibiotic, discharge, and/or death. Demographics included age, gender, height, and weight. Laboratory findings included kidney functions (serum creatinine, blood urea nitrogen), liver functions (alanine aminotransferase, aspartate aminotransferase, albumin,) and complete blood count.

Comorbidities, source of infection, and total duration of therapy are among the clinical data collected. Clinical signs and symptoms of infection are documented by the attending physician on the patient’s medical record. Laboratory values were pertinent to Acute Physiology and Chronic Health Evaluation II (APACHE II) calculations. It is a scoring system to assess disease severity based on the present physiologic measurements, age & preceding health conditions. The total number scored can help in the assessment of patients to determine the level as well as the degree of diagnostic & therapeutic intervention.

### Microbiological evaluation

Cultures and sensitivity tests from the suspected site of infection were withdrawn from all patients at baseline (before starting the antibiotic). Antibiotic susceptibility was carried out using agar diffusion and broth dilution method according to clinical and laboratory standards institute (CLSI) guidelines^18^.

### Clinical evaluation

Every day the patients’ clinical response was assessed by the attending physician and confirmed by a group of consultants assigned for daily medical rounds in the ICU through the close monitoring of clinical signs and symptoms of infection, white blood cell (WBC) count, and body temperature at baseline and at the termination of treatment. Clinical success was considered fulfilled if the following criteria were met: Resolution or improvement of clinical signs and symptoms caused by the infection, normalized WBCs, and body temperature, while clinical failure was defined as: persistent or worsening of any one of the clinical signs and symptoms caused by the infection, elevated WBCs and/or body temperature). Duration of therapy varied among the patients based on the clinical judgment and the time of reaching the predefined clinical success.

### Adverse effects evaluation

Any adverse events occurring during the whole duration of the study were recorded.

### Cost analysis

Total charges per stay (including both direct and indirect expenses acquired from the finance department) were recorded such as prices of supplies, preparation, administration, daily hospital stay cost, and nurse cost per hour. All costs were calculated in Egyptian Pound (LE) and the total was converted into United States Dollar (USD) based on the fiscal year 2018–2019, where the average expected exchange rate of the USD was 17.25 LE^19^.

### Outcomes

The primary efficacy end-point was the mean number of days to clinical success and the percentage of patients who were clinically cured after treatment. The secondary end-points included Intensive Care Unit length of stay (ICU LOS), mortality, readmission within 30 days, and cost-effectiveness analysis based on the mean number of days to clinical success.

### Statistical analysis

Sample size and power were calculated using G power 3.1.9.4 software. When using the results obtained from Grant et al.^20^ (Days to normalization of fever in the extended infusion group (1.2 ± 0.8 days) versus intermittent infusion group (2.4 ± 1.5 days), p = 0.012), the total sample size was 46 patients (23 in each group), assuming that α error probability = 5% and the power is 80%. Using SPSS (Statistical Package for the Social Sciences) version 23.0 to do the statistical analysis. Quantitative variables were reported as mean ± standard deviation (SD) or as median [interquartile range (IQR)]. The student’s t-test was applied several times to compare both groups. Categorical variables were reported as the number (%) of patients with the specific characteristic. Demographics and clinical characteristics of both arms were compared using the Mann–Whitney U-test. Categorical variables were compared using Chi-square or Fisher’s exact test. Statistically significant results are those of p-value < 0.05.

### Ethical approval

All named authors meet the International Committee of Medical Journal Editors (ICMJE) criteria for authorship for this article, take responsibility for the integrity of the work as a whole, and have given their approval for this version to be published.

### Consent to participate

Before participation, all guardians of eligible patients were informed about the study protocol and they provided the written informed consent.

## Results

### Study description

**Figure 1:**
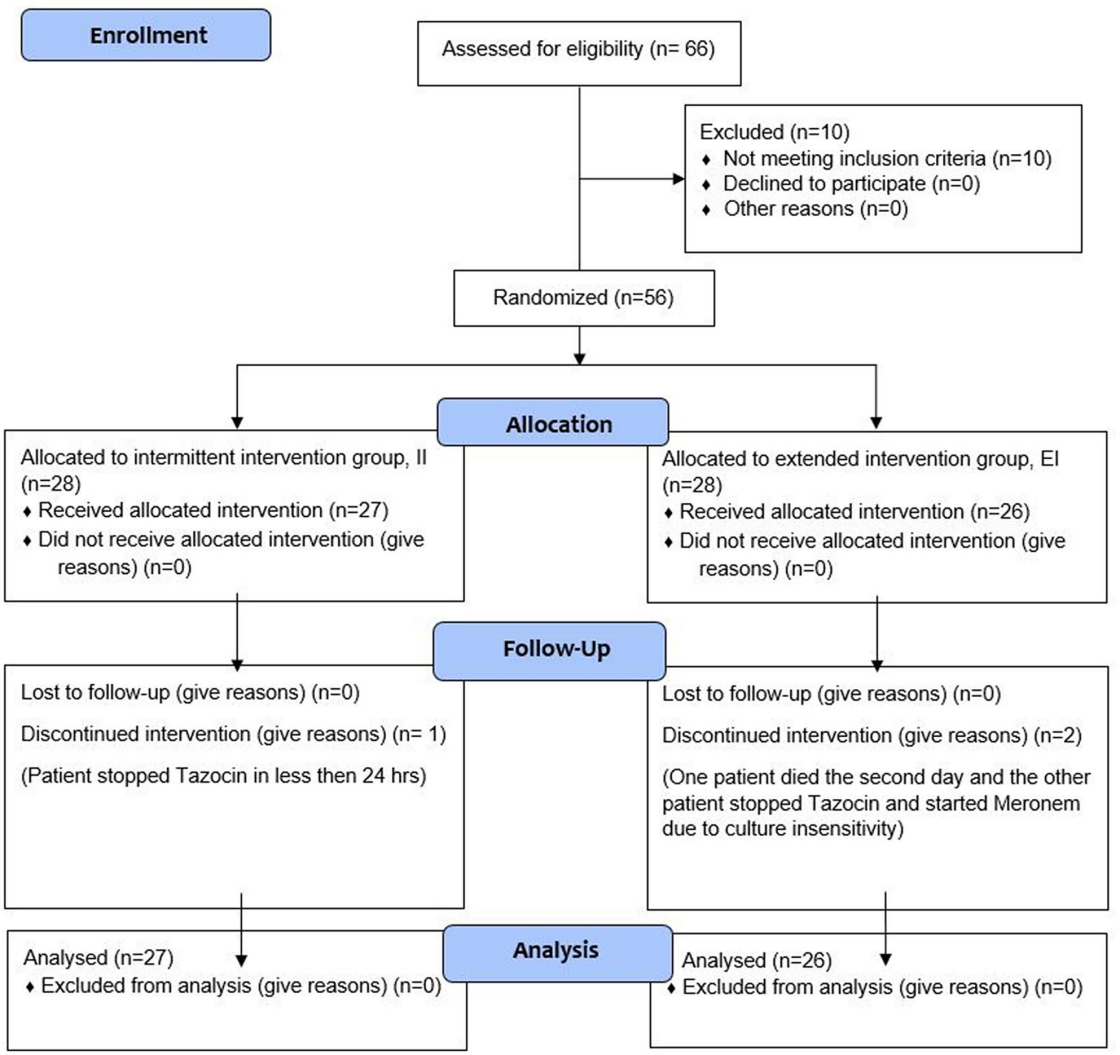
Study flowchart.

Sixty-six patients were assessed for eligibility. A total of 56 were included (10 patients were excluded for different reasons including 2 having cancer, 3 with renal impairment, and 5 due to their age). Patients were randomized into two groups: Group II: The intermittent Infusion group (28 patients) and Group EI: The extended infusion group (28 patients). In Group II, one patient was excluded after randomization due to discontinuation of the study drug after 24 h of initiation, and 2 patients were excluded in Group EI as one patient died the second day and the other patient stopped Tazocin and started Meronem due to culture insensitivity. The total number of patients who completed the study was 53 patients (27 in Group II and 26 in Group EI), Fig. 1. The baseline clinical characteristics and demographics of the study sample are presented in Table 1.

**Table 1:** Demographics and clinical characteristics of patients.

Characteristic	Intermittent (n = 27)	Extended (n = 26)	P value
**Age (years)**^**a**^			
(Mean ± SD)	53.0 ± 17.61	57.8 ± 15.35	0.291
**Sex, n (%)**^**b**^			
Male	14 (52%)	12 (46%)	0.678
Female	13 (48%)	14 (54%)	
Mechanical Ventilation, n (%)^b^	19 (70%)	18 (69%)	0.928
APACHE II score (Median & IQR)^c^	11 (7.0–16.0)	8.5 (6.75–13.25)	0.275
Diabetes, n (%)^b^	11 (41%)	10 (38%)	0.865
Smoking, n (%)^d^	4 (15%)	1 (4%)	0.172
SCR^a^ (Mean ± SD)	1.8 ± 0.93	1.1 ± 0.54	**0.002**
ALT^a^ (Mean ± SD)	90.0 ± 191.04	74.7 ± 117.13	0.727
AST^a^ (Mean ± SD)	127.0 ± 256.34	58.4 ± 61.10	0.183
WBCs (*10^9^ /L)^a^(Mean ± SD)	11.9 ± 5.81	11.3 ± 8.6	0.784
Temperature (^o^C ) ^a^ (Mean ± SD)	37.4 ± 0.78	37.2 ± 0.46	0.475
Positive cultures, n (%)^b^	12 (44.4%)	15 (57.7%)	0.414
**Infection source, n (%)**^**b,d**^			
Respiratory tract	18 (66.7)	15 (57.6)	0.577
Wound	1 (3.7)	0 (0)	1.000
Urinary tract	2 (7.4)	4 (15.3)	0.420
Skin or soft tissue	0 (0)	2 (7.6)	0.236
Abdominal	0 (0)	1 (3.8)	0.491
Blood	4 (14.8)	4 (15.3)	1.000
Respiratory and blood	2 (7.4)	0 (0)	0.491
Vasopressors	16 (59.3)	10 (38.5)	0.130
**Concomitant antibiotics, n (%)**^**b,d**^			
Vancomycin	14 (51.9)	4 (15.4)	0.005
Aminoglycosides	1 (3.7)	2 (7.7)	0.530
Clindamycin	1 (3.7)	2 (7.7)	0.530
Quinolones	12 (44.4)	15 (57.7)	0.335
Linezolid	0 (0)	2 (7.7)	0.142

All patients are Egyptians. Data are reported as mean ± SD, number (%) or median (interquartile range), *ALT* Alanine Transaminase, *AST* Aspartate Aminotransferase, *APACHE* II Acute Physiology and Chronic Health Evaluation, *SCR* Serum Creatinine, *WBCs *White blood cells. Results were compared using ^a^Independent T-test, ^b^chi-square test, ^c^Mann-Whitney U test, ^d^Fisher’s exact Test. P-value ≤ 0.05 is considered statistically significant.

Significant P-values are in bold.

Demographic and clinical characteristics were comparable in both groups. Similarly, as per diagnosis, patients were categorized among the different types of infection with no differences between groups. Respiratory tract infection was the predominant source of infection in both groups. Serum Creatinine was significantly more in the II group than in the EI group.

### Concomitant antibiotics

In the two groups, similar number of patients received concomitant antibiotics (n = 19, 70.4% in the II group *vs* n = 16, 61.5% in the EI group; p = 0.497). In both groups, the use of aminoglycosides, clindamycin, and quinolones was comparable. Vancomycin was the most concomitant antibiotic used specifically in II than in the EI Group (51.9% vs 15.4%, p = 0.005). Also, linezolid was used only in the EI group (2 patients).

### Primary and secondary clinical outcomes

In the present study as shown in Table 2, the clinical success was non-statistically different between both groups (22.2% in the II group & 19.2% in the EI group). There was no difference in mortality of participants in both groups (37% in the II group & 42% in the EI group). No significant differences in 30-day readmission were also noted in any of the two groups (26% in the II group &19% in the EI group). Patients stayed in the hospital for more days in the EI group but were not significantly different from the II group (19.1 ± 23.5 in the II group & 22.2 ± 15.79 in the EI group, p = 0.579). Days of therapy with piperacillin-tazobactam were different among the groups: 8.9 ± 3.57 for the II group versus 5.7 ± 2.07 for the EI group which is less than the other arm though.

**Table 2:** Clinical and microbiological outcomes.

Outcome	Intermittent (n = 27)	Extended (n = 26)	P-value
Clinical success, n (%)^b^	6 (22.2%)	5 (19.2%)	0.788^d^
LOS (days)^a^ (Mean ± SD)	19.1 ± 23.5	22.2 ± 15.79	0.579
30-day readmission, n (%)^b^	7 (26%)	5 (19%)	0.744
Mortality in hospital, n (%)^b^	10 (37%)	11 (42%)	0.695
Mean Duration of piperacillin/tazobactam (days)^a^ (Mean ± SD)	8.9 ± 3.57	5.7 ± 2.07	**0.0001**

Results are expressed as mean ± SD or number (%) and were compared using ^a^independent T-test, ^b^chi-square test, ^c^Mann- Whitney U test, ^d^ Fisher’s exact Test. P-value ≤ 0.05 is considered statistically significant.

*LOS* Length of Stay.

Significant P-values are in bold.

### Microbiological characteristics

Various types of cultures were withdrawn depending on the source of infection as presented in Table 3. More than one culture type could be withdrawn from the same patient. The four types of cultures withdrawn from patients were; sputum, blood, wound, and urine cultures. Major cultures withdrawn were sputum (63%) from the II group versus (77%) from the EI group. The following microorganisms were isolated from both groups: *Klebsiella pneumonia, Acinetobacter spp., Providencia spp., P. Aeruginosa, S. aureus, Enterobacter spp., and other gram-negative bacteria.* Positive cultures were comparable between the two groups except for urine cultures (25% in the II group versus 86% in the EI group).

**Table 3:** Identified organisms from cultures and their susceptibilities.

Variable	Intermittent (n = 27)	Extended (n = 26)	P-value
Sputum, n (%) ^a^	17 (63%)	20 (77%)	0.268
Positive cultures	10/17 (59%)	10 /20 (50%)	0.591
*K.pneumoniae*	1/10 (10%)	3/10 (30%)	
*Acinetobacter spp* *Providencia spp.*	1/10 (10%) NA	1/10 (10%) 1/10 (10%)	
*P.aeruginosa* *S.auerus* *Enterobacter spp.* *Other Gram* *Negative bacteria*	NA NA NA 8/10 (80%)	1/10 (10%) 1/10 (10%) 1/10 (10%) 2/10 (20%)	
Blood, n (%) ^b^	11 (41%)	9 (35%)	0.645
Positive cultures	2/11 (18%)	1/9 (11%)	0.651
*Other Gram* *negative bacteria*	2/2 (100%)	1/1 (100%)	
Wound, n (%) ^b^	6 (22%)	2 (8%)	0.139
Positive cultures	2/6 (33%)	2/2 (100%)	0.102
*S.auerus* *K.Pneumoniae* *Other Gram* *negative bacteria*	1/2 (50%) 1/2 (50%)	1/2 (50%) 1/2 (50%)	
Urine, n (%) ^b^	13 (48%)	7 (27%)	0.111
Positive cultures	4/13 (25%)	6/7 (86%)	0.019
*P.aeruginosa* *K.pneumoniae* *S.auerus* *Escherichia coli* *Other Gram* *negative bacteria*	1/4 (25%) NA NA NA 3/4 (75%)	NA 1/6 (16.6%) 1/6 (16.6%) 1/6 (16.6%) 3/6 (50%)	

Results are expressed as mean ± SD or number (%) and were compared using ^a^ chi-square test or ^b^ Fisher’s exact test where appropriate. P-value ≤ 0.05 is considered statistically significant.

### Safety analysis

The occurrence of adverse events is presented in Table 4. The adverse events observed were thrombocytopenia, hypokalaemia, hypernatremia, nephrotoxicity, and elevation in liver enzymes. Adverse events were comparable in both groups, except for aspartate aminotransferase and alanine aminotransferase which were significantly elevated in the II group compared to the EI group. Moreover, regarding patients with nephrotoxicity, 6 out of the 7 (85.7%) patients in the II were on vancomycin versus 4 out of the 6 (66.6%) patients in the EI group.

**Table 4:** Adverse events in both groups.

Outcome	Intermittent (n = 27)	Extended (n = 26)	P value
Thrombocytopenia (n%)^a^	8 (29.6)	11 (42.3)	0.336^a^
Hypokalaemia (n%)^a^	9 (33.3)	4 (15.4)	0.129
Hypernatremia (n%)^a^	4 (14.8)	2 (7.7)	0.413
Elevated ALT (n%)^a^	6 (22.2)	1 (3.8)	**0.048**
Increased AST (n%)^a^	9 (33.3)	1 (3.8)	**0.006**
Nephrotoxicity (n%)^a^	7 (25.9)	6 (23.1)	0.81

^a^Chi-square Test. *ALT* alanine aminotransferase, *AST* aspartate aminotransferase. Thrombocytopenia (Platelets < 150 *10^9^L), Hypokalaemia (Potassium < 3.5 mEq/L), Hypernatremia (Sodium > 145 mEq/L), Increased ALT and AST (> double baseline value). Nephrotoxicity was defined based on Kidney Disease: Improving Global Outcomes (KDIGO) and the Acute Kidney Injury Network (AKIN) i.e. A threshold increment of > 0.3 mg/dL in S.cr over 48-h^21^. P-value ≤ 0.05 is considered statistically significant.

Significant P-values are in bold.

### Pharmacoeconomic analysis

**Figure 2:**
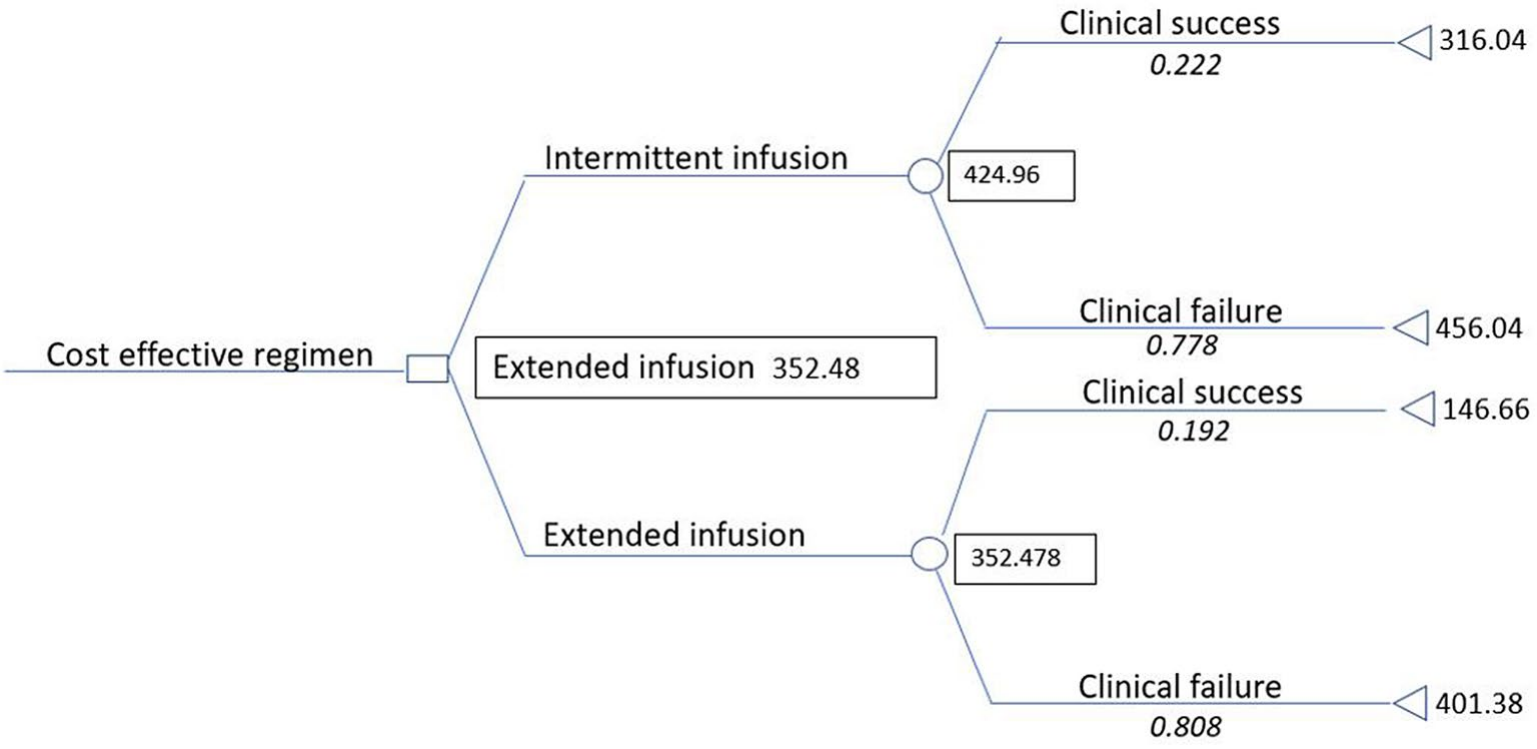
Decision tree results of drug acquisition economic analysis of intermittent infusion piperacillin-tazobactam compared with the extended infusion.

**Figure 3:**
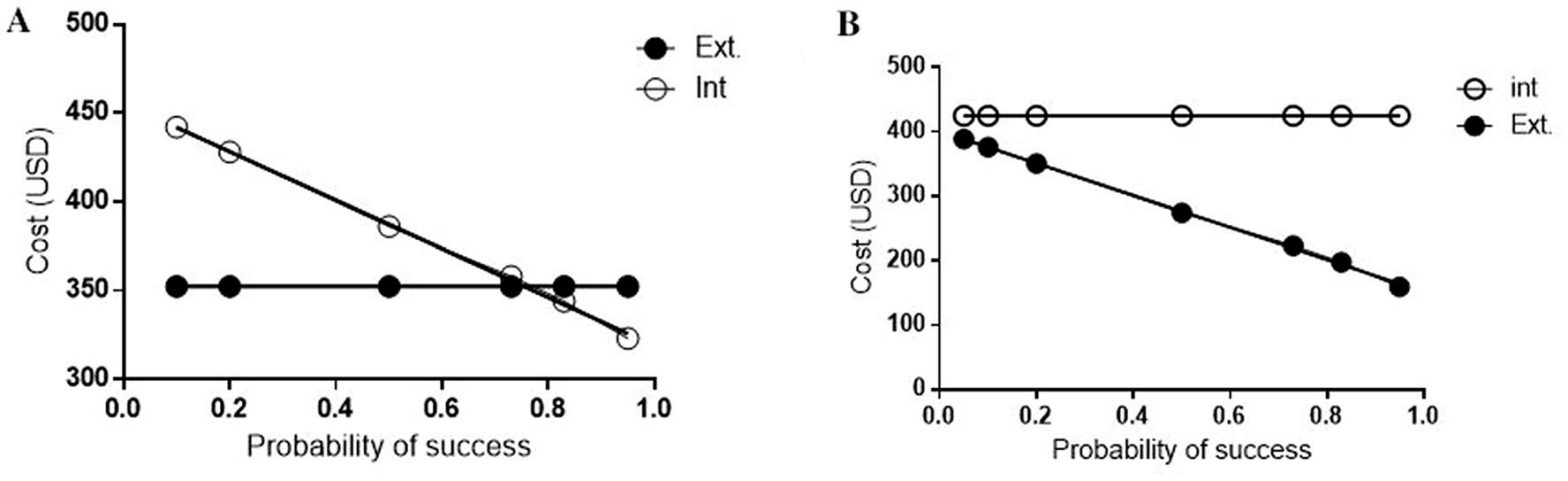
(**A**) One-way sensitivity analysis of extended infusion piperacillin-tazobactam compared with intermittent infusion, varying the probability of clinical success. The extended infusion was kept constant at 19.2% and intermittent infusion varied from 10–95%. (**B**) One-way sensitivity analysis of extended infusion piperacillin-tazobactam compared with intermittent infusion, varying the probability of clinical success. The clinical success of intermittent infusion was kept constant at 22.2% and extended infusion varied from 5–95%.

A pharmacoeconomic analysis was performed for costs from the health care provider perspective, comparing treatment with extended infusion *vs* intermittent infusion. The analysis encompassed all expenses directly related to antibiotic use: supplies, preparation, administration, daily hospital stay cost, acquisition prices of the antibiotic, and nursing time. Results of the cost analysis are provided in Table 5. There was a statistically significant difference in days of treatment success (8.5 ± 3.2) in the II group vs (4.6 ± 0.54) in the EI group, p = 0.026. Mean total costs for treatment success of extended infusion (146.66 ± 19.40) were significantly lower than for intermittent infusion (316.04 ± 103.83, p = 0.0061). The cost-effectiveness ratio, or cost/expected success, which is calculated as the cost of treatment divided by the probability of success, was also less for extended infusion compared with intermittent infusion ($1914.09 vs $1835.41, respectively). A folded-back decision tree is shown in Fig. 2. Costs of patients who failed or succeeded in the extended infusion were lower than that of the intermittent infusion. When the tree was folded back, the extended infusion regimen was still the more cost-effective: 352.48 $/patient compared with the intermittent infusion (424.96$/patient). Performing a one-way sensitivity analysis revealed that the probability of success of each treatment was independently varied (Fig. 3A,B).

**Table 5:** Comparing costs of both arms.

	Intermittent infusion (mean ± SD)	Extended infusion (mean ± SD)	P-value
Days of piperacillin-tazobactam therapy	8.9 ± 3.57	5.7 ± 2.07	**0.0001**
Days until treatment success	8.5 ± 3.2	4.6 ± 0.54	**0.026**
Total Costs ^a^	$424.93 ± 368.13 (n = 27)	$352.40 ± 187.59 (n = 26)	0.7875
Cost of treatment success	$316.04 ± 103.83 (n = 6)	$146.66 ± 19.40 (n = 5)	**0.0061**
Cost of treatment failure	$456.04 ± 339.73 (n = 21)	$401.38 ± 175.54 (n = 21)	0.5162
Cost-effectiveness ratio ^b^	$1914.09	$1835.41	

^a^Total cost in USD for preparations, supplies, drugs, and labor was obtained from the hospital during the year 2018–2019^b^. Cost-effectiveness ratio = mean Total costs/ success rate. P-value ≤ 0.05 is considered statistically significant.

Significant P-values are in bold.

When the extended infusion was kept constant at 19.2% and intermittent infusion varied from 10–95%. Extended infusion becomes the least costly regimen when intermittent infusion efficacy drops below 75%, Fig. 3A. When intermittent-infusion clinical success was kept constant at 22.2% and extended infusion varied from 5–95%, the economic decision favored the extended infusion regimen. Intermittent infusion remains the costliest regimen throughout the range of extended infusion clinical success, Fig. 3B.

## Discussion

The B-lactam antibiotics, including piperacillin/tazobactam, are among the first-line therapy used in critically ill patients because of their large antimicrobial spectrum and low toxicity. There is large evidence that extended infusion of B-lactam antibiotics improves outcomes because of time-dependent antibacterial activity compared with intermittent dosing^10,11^.

Based on our knowledge, this is the first study in the middle east to assess the use of conventional 30 min II of piperacillin/tazobactam versus the 4 h EI in critically ill patients with suspected or confirmed gram-negative infections from both the clinical and economic perspective. The study demonstrated similar clinical outcomes for the EI regimen compared with II favoring the trend of most studies done evaluating the two regimens^7,9,11,22^. Our study revealed that cost was reduced with the EI method of the administration going parallel to a large study done in a community teaching hospital in New Jersey^20^. Few studies evaluated the economic outcome of both regimens showing the same results as the present study^5,20,23^.

The results of the present study showed no statistically significant difference between the II and EI concerning clinical success (19.2% vs 22.2%, p = 0.788). Following a study done by Cotrina-Luque et al.^9^ in 11 Spanish hospitals comparing continuous infusion (given over 24 h) versus II (given over 30 min. every 8 h) of piperacillin/tazobactam in infections due to suspected pseudomonas aeruginosa. The latter study found both regimes equal in clinical success (p = 0.185). However, days of piperacillin/tazobactam therapy in EI were significantly lower than that of II (8.9 days in II vs 5.7 days in EI, p = 0.0001) as well as days to clinical success (8.5 days in II vs 4.6 days in EI, p = 0.026). Similarly, only one study done by Fan et al. when they compared the meantime to defervescence found that it was significantly reduced in the EI group (4 days in the EI group vs 6 days in the NEI group, p = 0.01)^24^.

On the other hand, a recent systematic review and meta-analysis evaluated articles published between 1998 to 2019 showed that there is a significant level of evidence that clinical outcome in critically ill patients is improved in patients receiving piperacillin-tazobactam via continuous/prolonged infusion^25^. Also, Yang et al. in their meta-analysis study showed a superior clinical success rate for the continuous infusion (being used over 24 h) than the conventional intermittent approach (over 30 min. three or four times daily)^4^.

The two meta-analyses used different characteristics of participants that were included in the study, the mode of administration of the piperacillin/tazobactam in an extended or intermittent form as well as the way of measuring the primary outcome. All three explain why those studies have led to a different outcome than the current study.

The presence of a wide variety of infections is being explained by the nature of the patients ‘conditions being all critically ill patients. However, the most prevalent type of infection was respiratory infection similar to what was found by Cotrina-Luque et al. which explains the reason for both having the same clinical success outcome^9^.

In the present study, ICU mortality was comparable in both treatment arms 37% in II vs 42% in EI). Similarly, Gonçalves-Pereira et al. in a multicenter propensity-matched analysis compared the ICU mortality rate between both II and EI piperacillin/ tazobactam and showed a non-significant difference between the two groups (20.2% in II vs 23.7% in EI, p = 0.512)^10^.

Also, a prospective clinical trial demonstrated a similar 14-day mortality rate between the two regimens as well (p = 0.29)^24^. However, The systematic review is done by Yang et al., where five randomized studies and nine observational studies were included, showed a lower mortality rate (OR 0.67, 95% EI 0.50–0.89, p = 0.005) for the extended infusion than the conventional intermittent approach^4^. That difference in the mortality outcome being variable between studies could be related to the health conditions of the patients including their age and demographic data. However, to identify any group of patients who had a significant noteworthy health condition from the beginning, we stratified the patients according to their APACHE score and found no significant difference as was done in the study of Bao et al. as well^5^.

Searching for reasons that lead to the high percentage of death and failure in the present study being 77.8% in the II group and 80.8% in the EI group, we observed that the high prevalence of resistant strains according to the hospital’s antibiogram could be the main reason of that failure. High resistance to the piperacillin/tazobactam could be the reason behind the high percentage of treatment failure in both groups.

The present study showed statistically indifferent 30-day readmission and ICU length of stay between the two arms. Winstead EM et al. conducted a retrospective cohort study in a 433-bed hospital comparing 3 h extended infusion to the conventional intermittent regimen resulted in a significant reduction in the 30-day readmission outcome similarly (p = 0.002) which could be due to the different mode of administration^8^.

In the current study, almost all adverse events reported were the same in both arms, whereas, the II group showed a higher statistically significant increase in ALT and AST than with the EI regimen. As it is difficult to associate the increase in liver enzymes to the piperacillin/tazobactam regimen, we assume that this increase in liver enzymes in the EI regimen arm is most probably related to the specific patient’s clinical status and concomitant drugs (acetaminophen, NSAIDs, ACE inhibitors, statins) rather than the EI regimen itself. Moreover, it was noted in some studies that co-administrating piperacillin/tazobactam antibiotic with vancomycin is a possible risk that may enhance the incidence of nephrotoxicity^26–28^. Accordingly, we compared the incidence of nephrotoxicity in patients who were on vancomycin co-administered with the piperacillin/tazobactam in both groups. The results showed a possible decrease in the incidence of nephrotoxicity when using the EI versus the II (6 out of 14 patients who were on vancomycin in the II group experienced nephrotoxicity versus none out of 4 patients in the EI group). However, this finding may need further investigation due to the small sample size.

The results of this study showed that piperacillin/tazobactam extended infusion was superior to intermittent infusion regarding cost-effectiveness ratio ($1835.41 and $1914.09/expected success, respectively). The duration of antimicrobial therapy directly affected the total cost of therapy, where the mean cost of days until treatment success was $316.04 in the II group versus $146.66 in the EI group, p = 0.0061. This was demonstrated in a similar analysis of the costs done by Grant EM et al. that showed that extended infusion was less costly than intermittent infusion (p = 0.028)^20^.

Also, Brunetti et al. in their study evaluating the medical and financial impact of extended infusion piperacillin/tazobactam in a community medical center, concluded that it is safe and associated with significant cost savings^3^. Moreover, in a randomized controlled trial by Bao et al. carried out in China, the authors found fewer costs per patient about $430.32 in extended infusion versus II^5^. On the other hand, only one retrospective cohort study done by Winstead et al. showed no statistical difference between the total admission cost of both arms^8^.

The main limitation of this study is the enrolment from a single center and that most of the patients started the antibiotic empirically before organisms were identified. Also, many cultures were negative despite clinical evidence of infection which resulted in the unavailability of microbiologic confirmations of bacterial infections and susceptibility data in some patients. Accordingly, the statistically significant differences in the percentage of positive urine cultures in the intermittent versus the extended group may probably be a consequence of this limitation. However, only one patient with positive urine culture in each group had clinical success. Hence, this is unlikely to have influenced the results.

Moreover, concomitant glycopeptides (mainly vancomycin) were used extensively in the II group more than in the EI group (p = 0.005). Although, vancomycin was added empirically to cover methicillin-resistant staphylococcus aureus which is not covered by piperacillin/tazobactam, however, this probably didn’t affect the results since most patients with positive cultures had gram-negative infections and both arms had comparable clinical success.

## Conclusion

The more cost-effective regimen of piperacillin/tazobactam is the EI compared to the II in suspected or proven infections with gram-negative bacteria in critically ill patients. Both regimens proved to have the same clinical and microbiological outcomes.

## Data Availability

The datasets generated during and/or analyzed during the current study are available from the corresponding author on reasonable request.
